# Supplementary material for: DiMeLo-cito: a one-tube protocol for mapping protein-DNA interactions reveals CTCF bookmarking in mitosis
Source: bioRxiv. 2025 Mar 14:2025.03.11.642717. Preprint. [Version 1] doi: 10.1101/2025.03.11.642717 (PMC11952428; doi:10.1101/2025.03.11.642717)
Supplement: Supplement 1 [file NIHPP2025.03.11.642717v1-supplement-1.pdf]

bioRxiv preprint doi: https://doi.org/10.1101/2025.03.11.642717; this version posted March 14, 2025. The copyright holder for this preprint (which was not certified by peer review) is the author/funder, who has granted bioRxiv a license to display the preprint in perpetuity. It is made available under aCC-BY 4.0 International license.

Supplemental Figure

Figure 1 S1

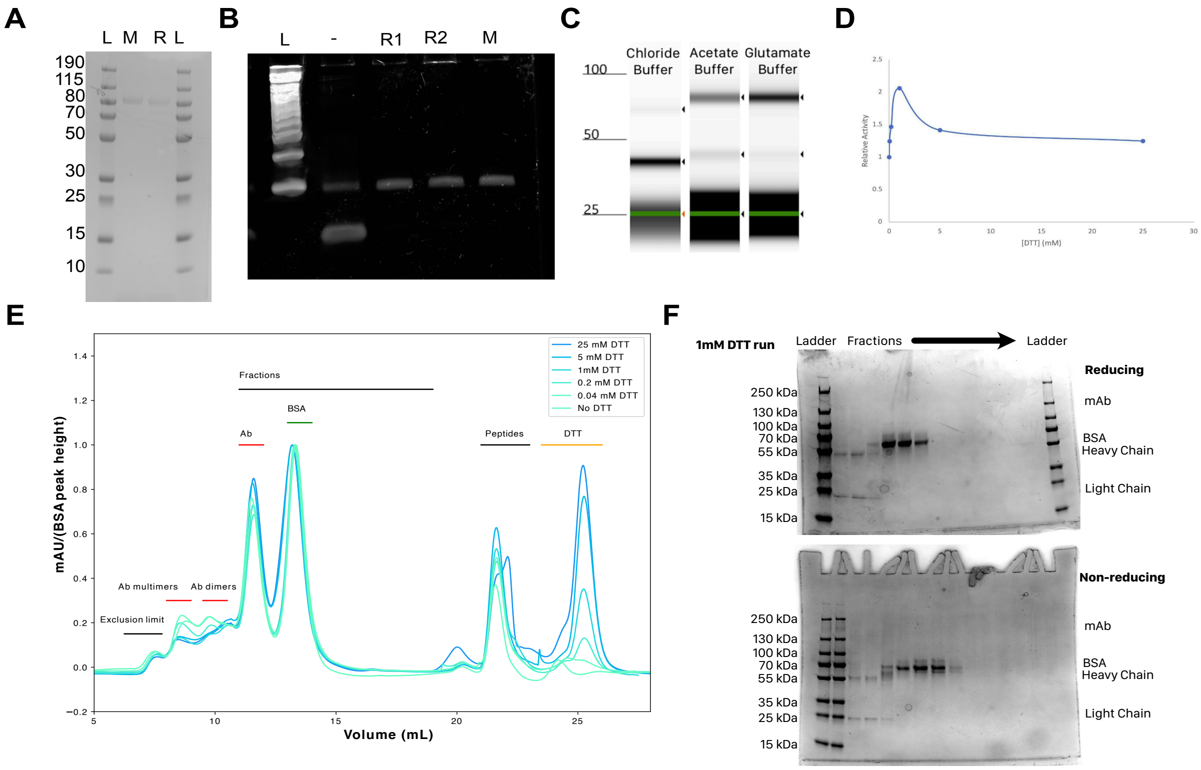

Figure 1 S1. Optimization of Hia5 reaction conditions. A. SDS-PAGE of purified MBP-anti-mouse-Hia5 (M) and MBP-anti-rabbit-Hia5 (R). **B.** Polyacrylamide gel of a restriction protection methyltransferase assay with DpnII for MBP-anti-mouse-Hia5 (M) and two preparations of MBP-anti-rabbit-Hia5 (R1/R2) in glutamate buffer. Uncut fragments indicate higher degrees of methylation. **C.** Electropherogram from a Tapestation D1000 showing a restriction protection assay using MBP-anti-rabbit-Hia5 in DiMeLo activation buffer with the indicated counterion. **D.** Relative protection of DNA in a restriction protection assay using chloride buffer with increasing concentrations of DTT. **E.** Chromatogram at A280 from injections of anti-CTCF antibody onto an S200 increase 10/300 size exclusion column after incubation at 37C for 1 hour with increasing concentrations of DTT. Locations of the eluting species are marked above peaks. **F.** Reducing and Non-reducing SDS-PAGE of fractions eluting from size exclusion chromatography of Antibody incubated with 1mM DTT prior to injection.

bioRxiv preprint doi: https://doi.org/10.1101/2025.03.11.642717; this version posted March 14, 2025. The copyright holder for this preprint (which was not certified by peer review) is the author/funder, who has granted bioRxiv a license to display the preprint in perpetuity. It is made available under aCC-BY 4.0 International license.

Figure 1 S2

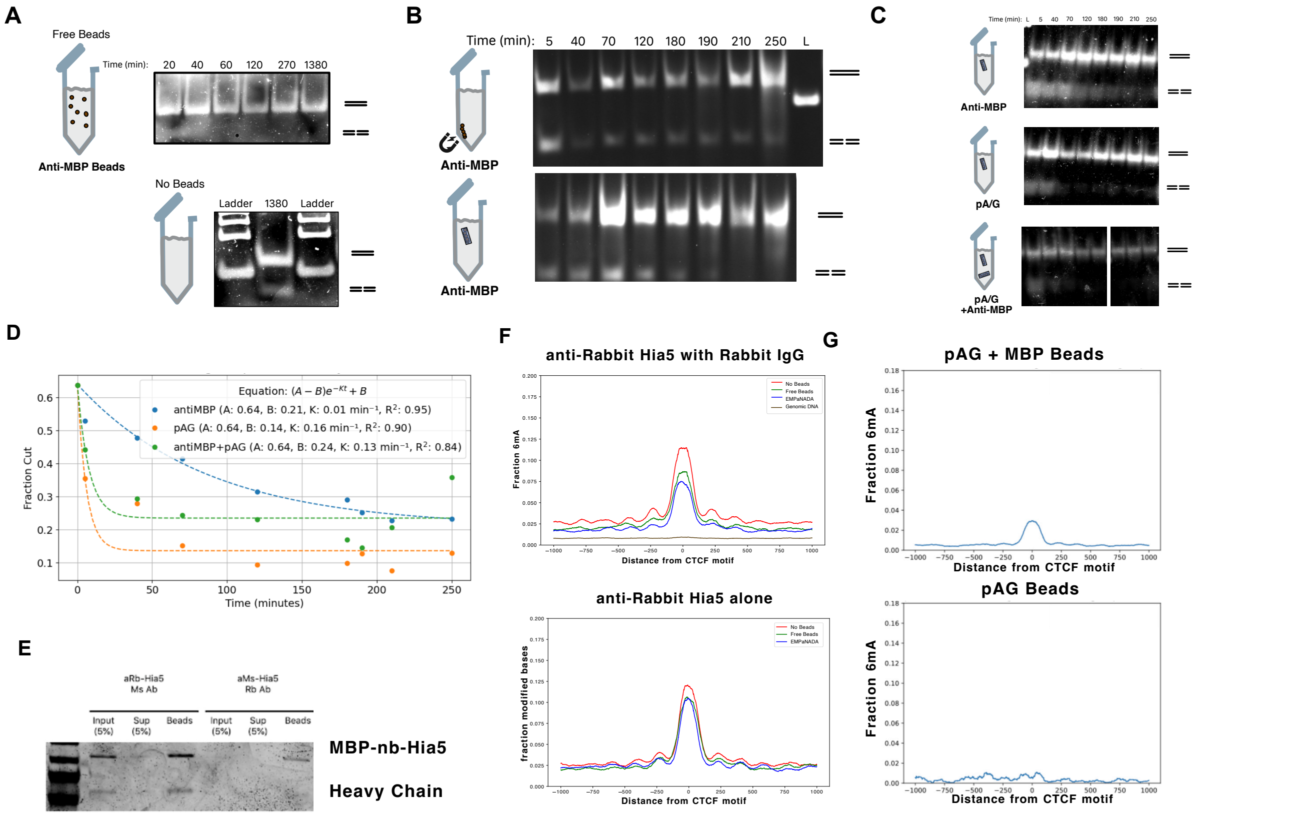

Figure 1 S2. Optimization of free-enzyme depletion. A. Kinetics of antibody enzyme complex depletion with free beads. Anti-MBP beads were incubated with precomplexed antibody-Hia5 complexes (1:3) at room temperature for 5 minutes. Supernatants retrieved at the indicated time were then subjected to a restriction enzyme methyltransferase assay using DpnI where higher amounts of cutting indicate more methyltransferase activity. **B.** Kinetics of antibody enzyme complex depletion by restriction enzyme methyltransferase assay with DpnI comparing incubation with paramagnetic anti-MBP beads enclosed in an EMPaNADA or retained against the wall of the tube with a magnet using a DpnI. **C.** Kinetics of antibody enzyme complex depletion by restriction enzyme methyltransferase assay comparing incubation with a single Anti-MBP EMPaNADA, a single pA/G EMPaNADA, or both a single anti-MBP and pA/G EMPaNADA. **D.** Fits to a single exponential decay for the depletion reactions in C. **E.** Precipitation of the indicated nanobody-Hia5 construct onto pA/G beads. Samples were also incubated with a nonbinding antibody as a positive control. **F.** DiMeLo-cito in GM24385 LCs with pA/G bead EMPaNADAs using anti-rabbit-Hia5 in the presence or absence of control antibody sequenced to ~1X coverage. Profile plots centered at CTCF peaks were generated with a 50 bp smoothing window. G. DiMeLo-cito using anti-rabbit-Hia5 with control antibody and either a single pA/G EMPaNADA or a pA/G and anti-MBP EMPaNADA. Samples were sequenced to ~0.1X coverage and profile plots centered at CTCF peaks were generated with a 50 bp smoothing window.

bioRxiv preprint doi: https://doi.org/10.1101/2025.03.11.642717; this version posted March 14, 2025. The copyright holder for this preprint (which was not certified by peer review) is the author/funder, who has granted bioRxiv a license to display the preprint in perpetuity. It is made available under aCC-BY 4.0 International license.

Figure 1 S3

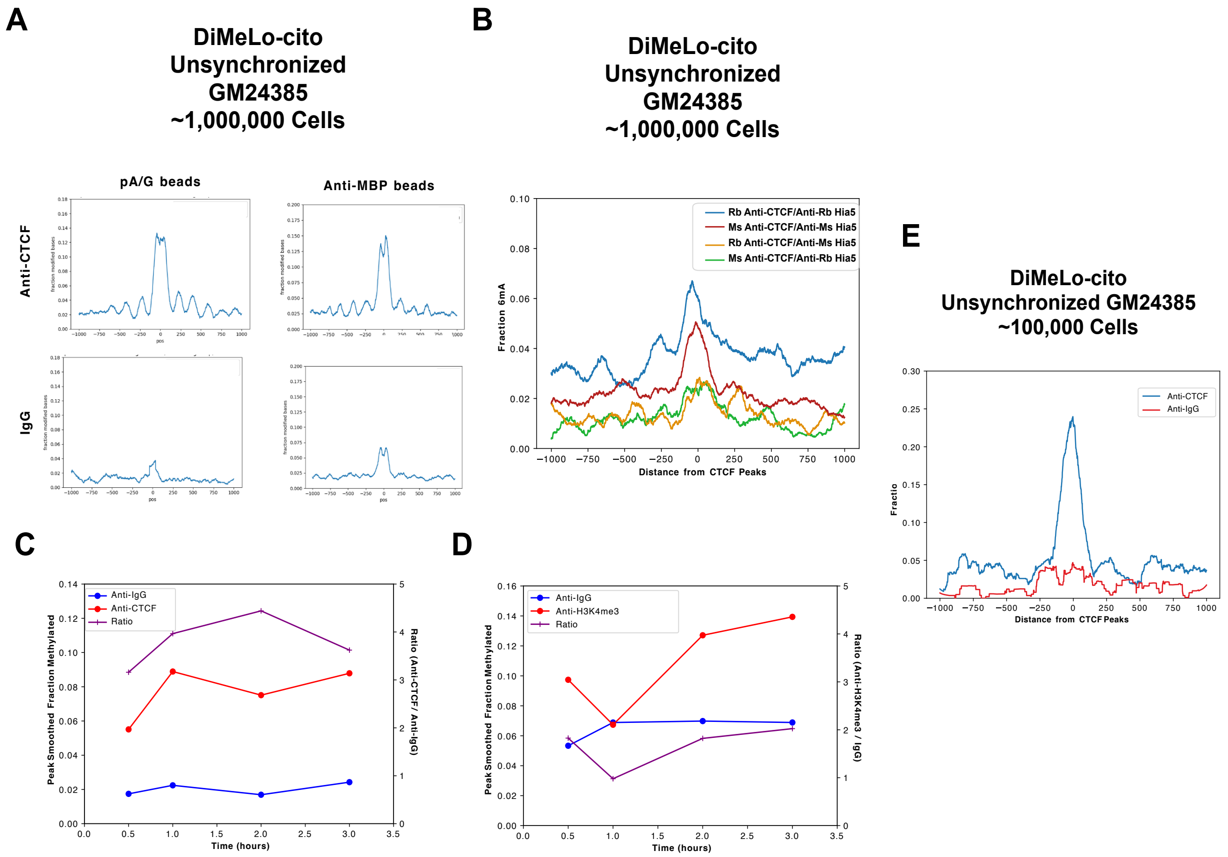

Figure 1 S3. Optimization of DiMeLo-cito. **A.** Comparison of DiMeLo-cito using CTCF targeting or non-targeting with 1 million cells using pA/G or anti-MBP paramagnetic beads sequenced to ~0.1X coverage. **B.** Comparison of DiMeLo-cito using rabbit or mouse CTCF targeting antibody combined with cognate or noncognate antibody. Samples were sequenced to ~0.1X coverage and profile plots centered at CTCF peaks were generated with a 50bp smoothing window. **C.** Kinetics of methyladenine deposition by anti-rabbit-Hia5 during DiMeLo-cito at CTCF sites using CTCF-targeting or non-targeting antibody. Each point is the peak intensity from profile plots centered at CTCF sites from a single DiMeLo-cito reaction incubated for the indicated time during the activation step. Each reaction was sequenced to ~0.1X coverage. **D.** Kinetics of methyladenine deposition by anti-rabbit-Hia5 during DiMeLo-cito at the top quartile of transcription start sites by RNA-seq using H3K4me3-targeting or non-targeting antibody. Each point is the peak intensity from 50 bp smoothed profile plots centered at H3K4me3 sites from a single DiMeLo-cito reaction incubated for the indicated time during the activation step. Each reaction was sequenced to ~0.1X coverage. **E.** DiMeLo-cito targeting CTCF or a nontargeting control performed with 100,000 cells. Plot is smoothed with a 50 bp sliding window.

bioRxiv preprint doi: https://doi.org/10.1101/2025.03.11.642717; this version posted March 14, 2025. The copyright holder for this preprint (which was not certified by peer review) is the author/funder, who has granted bioRxiv a license to display the preprint in perpetuity. It is made available under aCC-BY 4.0 International license.

Figure 1 S4

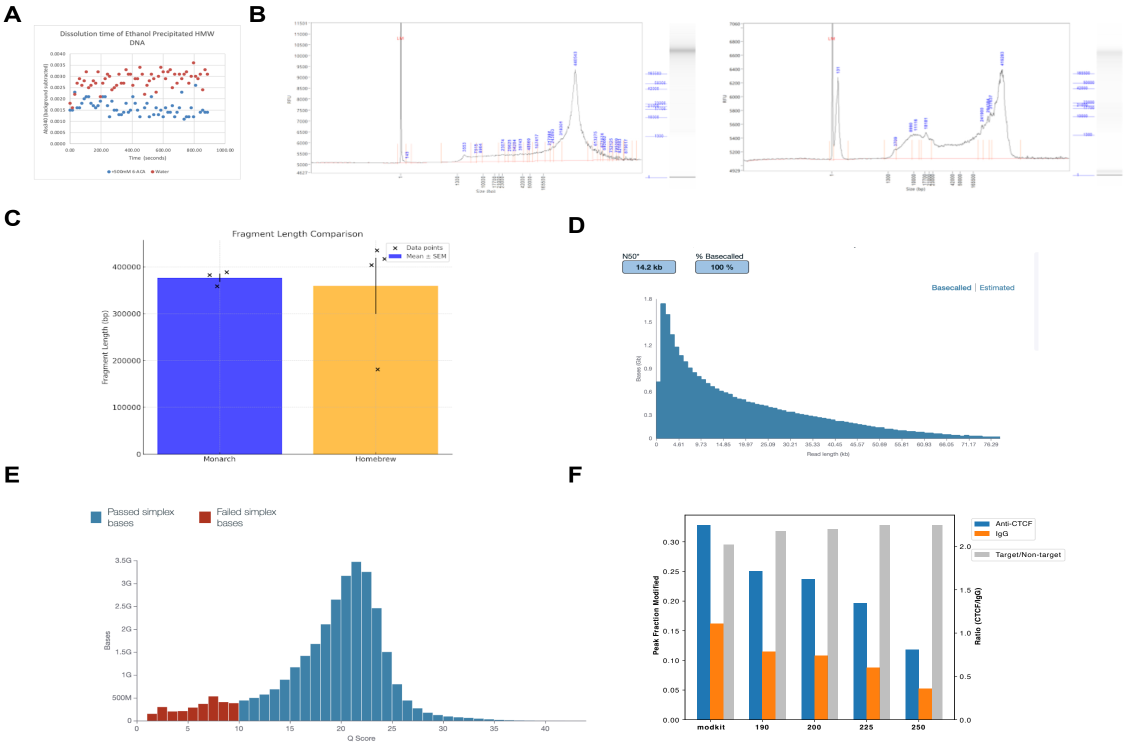

Figure 1 S4. Properties of DiMeLo-cito DNA extraction and sequencing. **A.** Dissolution kinetics of precipitated ultra high molecular weight genomic DNA extracted from HeLa cells monitored by light scattering at 340 nm. The time course compares incubation at room temperature with nuclease free water or with 0.5M 6-ACA pH 9.0. **B.** Screenshots from Femto Pulse runs of DiMeLo-cito samples extracted using NEBs Monarch HMW genomic DNA kit or using an in-house method (see methods). **C.** Median fragment length distributions from samples extracted using the NEB kit or in-house (homebrew) method. **D.** Fragment length distributions from library preparation by ligation sequencing of HMW DNA extracted using the in-house method and sheared to generate smaller fragments. Sequencing data from this run was used in the generation of the 30X coverage dataset presented in Figure 1**. E.** PHRED Q-score distributions of basecalls using the super accurate modified base calling models from the same run as D. **F.** Plots of methyladenine enrichment from DiMeLo-cito at CTCF sites using CTCF-targeting or nontargeting antibody and the ratio of the two at various modification confidence thresholds. The x axis values correspond to the chosen minimal MI cutoff score to be considered methyladenine or a cutoff score automatically chosen from the data by modkit within the dimelo package.

bioRxiv preprint doi: https://doi.org/10.1101/2025.03.11.642717; this version posted March 14, 2025. The copyright holder for this preprint (which was not certified by peer review) is the author/funder, who has granted bioRxiv a license to display the preprint in perpetuity. It is made available under aCC-BY 4.0 International license.

Figure 2 S1

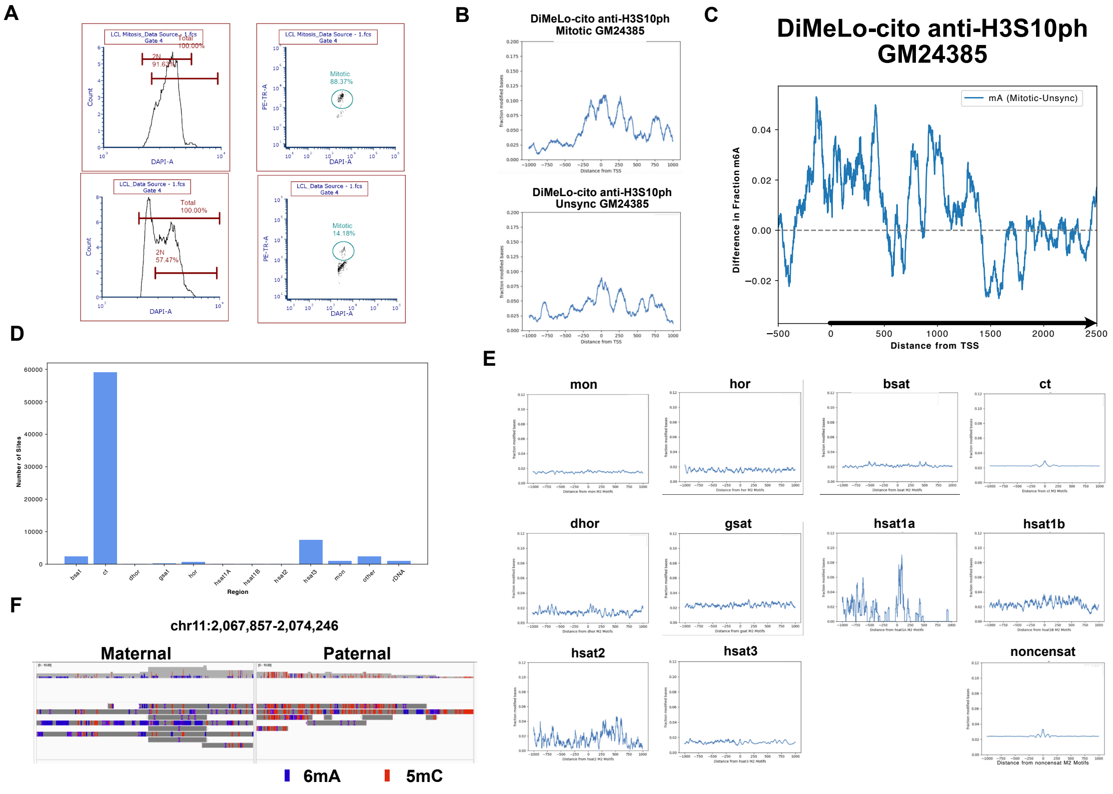

Figure 2 S1. Validation of mitotic enrichment and distributions of centromeric CTCF in GM24385 LCs by DiMeLo-cito. A. Flow cytometry quantifying the fraction of cells past S phase by DAPI staining (left) and the fraction of cells actively in mitosis (right) by IF staining of H3S10ph. B. Enrichment profile plots of methyladenine at active transcription sites using H3s10ph targeted DiMeLo-cito in either mitotic (top) or unsynchronized GM24385 LCs (bottom) both smoothed with a 50 bp sliding window. **C.** Difference plot of profiles in B. **D.** Instances of CTCF M2 motifs identified by fimo in different centromere/satellite (censat) DNA annotated regions in the CHM13v2.0-T2T genome. **E.** Profile plots with 50bp smoothing of methyladenine at M2 motifs separated by censat annotations as well as all noncensat sites in the CHM13v2.0-T2T genome. **F.** Screenshot from IGV read browser at the H19 imprinting control region of anti-CTCF DiMeLo-cito in mitotic GM24385 LCs aligned to the HG002v1.1 genome.

bioRxiv preprint doi: https://doi.org/10.1101/2025.03.11.642717; this version posted March 14, 2025. The copyright holder for this preprint (which was not certified by peer review) is the author/funder, who has granted bioRxiv a license to display the preprint in perpetuity. It is made available under aCC-BY 4.0 International license.

Figure 2 S2

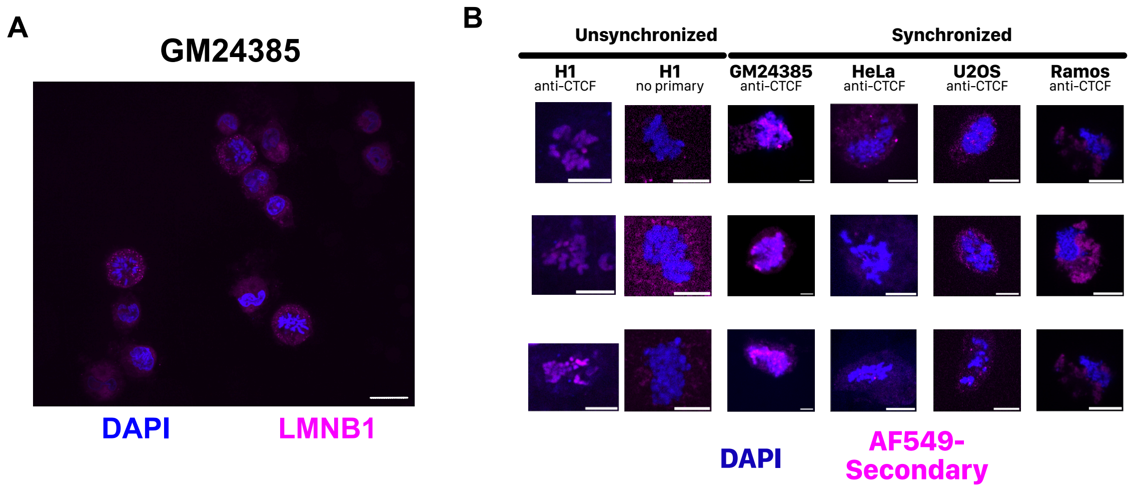

Figure 2 S2. Variation in mitotic chromosome enrichment of anti-CTCF staining. A. Confocal imaging of mitotic synchronized GM24385 stained for the lamina. Scale bar corresponds to 15 µm. **B.** Confocal imaging of CTCF-stained or unstained IF samples in various unfixed cell lines chemically synchronized in mitosis or unsynchronized. Scale bar corresponds to 15 µm.
